# Long-Term Pain Treatment Did Not Improve Sleep in Nursing Home Patients with Comorbid Dementia and Depression: A 13-Week Randomized Placebo-Controlled Trial

**DOI:** 10.3389/fpsyg.2018.00134

**Published:** 2018-02-13

**Authors:** Kjersti M. Blytt, Bettina Husebo, Elisabeth Flo, Bjørn Bjorvatn

**Affiliations:** ^1^Department of Global Public Health and Primary Care, University of Bergen, Bergen, Norway; ^2^Centre for Elderly and Nursing Home Medicine, University of Bergen, Bergen, Norway; ^3^Norwegian Competence Center for Sleep Disorders, Haukeland University Hospital, Bergen, Norway; ^4^Department of Nursing Home Medicine, Bergen, Norway; ^5^Department of Clinical Psychology, University of Bergen, Bergen, Norway

**Keywords:** sleep, nursing home, actigraphy, pain treatment, depression, pain

## Abstract

**Objective:** Previous research indicates that pain treatment may improve sleep among nursing home patients. We aimed to investigate the long-term effect of pain treatment on 24-h sleep patterns in patients with comorbid depression and dementia.

**Design:** A 13-week, multicenter, parallel-group, double-blind, placebo-controlled randomized clinical trial conducted between August 2014 and September 2016.

**Setting:** Long-term patients from 47 nursing homes in Norway.

**Participants:** We included 106 patients with comorbid dementia and depression according to the *Mini Mental Status Examination (MMSE)* and the *Cornell Scale for Depression in Dementia (CSDD).*

**Intervention:** Patients who were not using analgesics were randomized to receive either paracetamol (3 g/day) or placebo tablets. Those who already received pain treatment were randomized to buprenorphine transdermal system (maximum 10 μg/h/7 days) or placebo transdermal patches.

**Measurements:** Sleep was assessed continuously for 7 days by actigraphy, at baseline and in week 13. Total sleep time (TST), sleep efficiency (SE), sleep onset latency (SOL), wake after sleep onset (WASO), early morning awakening (EMA), and number of wake bouts (NoW) were evaluated. In addition, daytime total sleep time (DTS) was estimated. Pain was assessed with *Mobilization-Observation-Behavior-Intensity-Dementia-2 Pain Scale* (MOBID-2).

**Results:** The linear mixed model analyses for TST, SE, SOL, WASO, EMA, NoW and DTS showed no statistically significant differences between patients who received active pain treatment and those who received placebo. *Post hoc* subgroup analyses showed that there were no statistically significant differences between active treatment and placebo from baseline to week 13 in patients who were in pain (MOBID-2 ≥ 3) at baseline, or in patients who had poor sleep (defined as SE < 85%) at baseline. Patients who received active buprenorphine showed an increase in TST and SE compared to those who received active paracetamol.

**Conclusion:** The main analyses showed that long-term pain treatment did not improve sleep as measured with actigraphy. Compared to paracetamol, TST and SE increased among patients who received buprenorphine. This could indicate that some patients had beneficial effects from the most potent pain treatment. However, based on the present findings, long-term pain treatment is not recommended as a strategy to improve sleep. Clinical Trial https://clinicaltrials.gov/ct2/show/NCT02267057.

## Introduction

Approximately 46.8 million people worldwide suffer from dementia – a number estimated to reach 131.5 million by 2050 (Prince et al., 2016). In nursing homes (NH), 50–80% of patients have dementia (Helvik et al., 2015; Blytt et al., 2017b), a neurodegenerative condition that results in the decline of physical and cognitive functions (Cricco et al., 2001). Sleep disturbances are common among NH patients with dementia, with prevalence ranging from 24.5% (Moran et al., 2005) to 60% (Neikrug and Ancoli-Israel, 2010; Ownby et al., 2014; Peter-Derex et al., 2015). Dementia may induce pathophysiological changes in the brain, which can interfere with the maintenance of normal sleep (Moran et al., 2005; Neikrug and Ancoli-Israel, 2010). Previous studies have reported that people with dementia have more disturbed sleep than do patients without dementia (Pat-Horenczyk et al., 1998). It is further noteworthy that previous research indicates that NH patients with dementia are rarely asleep or awake for a full hour in the 24-h cycle (Jacobs et al., 1989). NH patients may suffer dramatic consequences from sleep disturbances, for instance by increasing the risk of falls and hip fractures (Stone et al., 2004; Morley, 2013; Widera, 2013) and decreasing survival (Dew et al., 2003). Furthermore, sleep disturbances contribute to impaired daytime functioning (Cricco et al., 2001).

Several factors contribute to sleep disturbances among NH patients, including pain (Chen et al., 2011; Flo et al., 2017) and depression (Giron et al., 2002). Approximately 20–30% of NH patients have depression, a disorder highly associated with sleep disturbances (Potter and Steffens, 2007). Depression is a common mental disorder, of which central symptoms are low mood and low or loss of ability to experience pleasure (American Psychiatric Association, 2013). Around 50% of the people with Alzheimer disease experience depression during the course of the disease (Lyketsos and Olin, 2002). Furthermore, nearly 60% of NH patients experience pain every day (Husebo et al., 2010). Pain is an unpleasant sensory and emotional experience (Onen et al., 2005) and represents an important cause for poor sleep among NH patients (Morley, 2013). Patients with dementia may have reduced capacity to express symptoms, e.g., pain or sleep disturbances. For this reason, it is essential that NH staff strives to evaluate symptoms through appropriate methods. Research suggests that pain and depression share common signal pathways and neurotransmitters, which implies that they may be responsive to comparable treatments. This intimate relationship is denoted the pain-depression dyad (Chopra and Arora, 2014).

Medications such as atypical antipsychotics, benzodiazepines and other GABAergic drugs are often sought to alleviate sleep problems in people with dementia (McCleery et al., 2016). However, previously conducted studies indicate that the source of sleep problems might be changes in the brain caused by dementia (Montplaisir et al., 1995, 1998; Kinnunen et al., 2017). Therefore, the efficacy of treatment with various drugs in this patient group is highly questionable (McCleery et al., 2016). Meanwhile, a study conducted by Husebo et al. (2013) found that a stepwise protocol for treating pain improved mood and sleep, as measured with the *Neuropsychiatric Inventory – Nursing Home version (NPI-NH)*, in people with advanced dementia and agitation. Furthermore, in a recently published randomized controlled trial, based on the same dataset and respondents as the present work, we found that compared to placebo, pain treatment improved sleep after 1 week of treatment (Blytt et al., 2017a). In the present study, we aim to investigate the long-term effect of pain treatment on sleep in patients with comorbid dementia and depression. In light of the results from Blytt et al. (2017a), we hypothesized that long-term pain treatment would improve sleep after 13 weeks in patients with comorbid dementia and depression.

In additional *post hoc* subgroup analyses, we further aimed to investigate if improvement of sleep from pain treatment was larger in patients who were in pain at baseline, defined as *Mobilization-Observation-Behavior-Intensity-Dementia-2 Pain Scale* (MOBID-2) score ≥ 3, than in those who were not. In addition, we aimed to investigate the effects of pain treatment on different sleep outcomes for patients with poor sleep at baseline, defined as sleep efficiency (SE) < 85%. In the last analysis, we aimed to examine if there were any differences within the active treatment group, i.e., between patients receiving active buprenorphine and active placebo, respectively.

## Materials and Methods

The study is based on an actigraphy subproject in the 13-week, multicentre, parallel-group, double-blind, placebo-controlled randomized trial “*Efficacy of Pain Treatment on Depression in Patients with Dementia – A Randomized Clinical Trial of Efficacy: DEP.PAIN.DEM.*” The study was conducted from August 2014 to September 2016, in Norway. We included 47 NHs from 11 municipalities, located in both urban and rural areas in Norway. In the present study, we used sleep data collected in the week before treatment commenced (baseline) and in week 13 of the treatment/placebo period.

### Participants and Procedures

**FIGURE 1:**
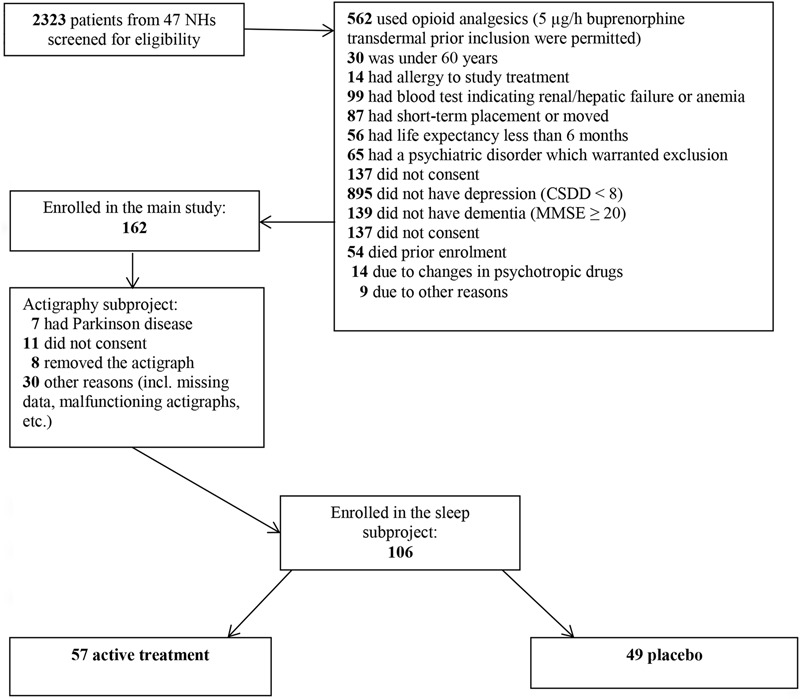
Flow chart screening and inclusion (reprinted from Blytt et al., 2017a).

Data collection was led by two researchers who enrolled NHs through direct contact with NH management. If the management agreed to be part of the project, the researchers were given access to patient medical journals to perform a pre-screening review. If there were no recent blood analyses (electrolytes, hemoglobin, serum creatinine, and serum alanine aminotransferase) available, new were requisitioned. In order to be included, patients had to be ≥60 years, long term NH patients with >4 weeks of stay, dementia as indicated by *Mini Mental State Examination* (MMSE ≤ 20) and depression as indicated by the *Cornell Scale for Depression in Dementia* (CSDD ≥ 8). Patients were excluded from the study if they had severe medical disease that could interfere with study participation, were using any opioid analgesic (except buprenorphine 5 mcg/h), did not want to wear an actigraph, were immobile or had involuntary movements. Inclusion and exclusion criteria are covered extensively in Blytt et al. (2017a). The patient was reassessed after written consent was given, and a drop from ≥8 to ≥6 in CSDD was permitted between screening and baseline. In addition to all of the inclusion and exclusion criteria, the treatment needed to be approved by the physician responsible for the patient (see the flow chart in **Figure 1** for an overview of enrolment and reasons for exclusion). We assessed the patients with the same measurements at baseline and in week 13 of the treatment period.

A stepwise protocol, with a fixed-dose regimen, for treating pain was used in the study period (see **Table 1**). Patients were allocated either to a paracetamol group or to a buprenorphine group and randomized to receive active treatment or placebo. If a patient showed any signs of not tolerating the treatment (e.g., headache, dizziness or nausea), needed to change medical treatment, or there was anything else conflicting with the patient taking part in the study, the patient was withdrawn from the study and the reason was recorded. During the study period, all patients continued their usual medical treatment.

**Table 1:** Overview of how patients were assigned to treatments.

Step	Regular analgesic treatment	Randomly assigned to either:	Dose
1	No analgesics or paracetamol ≤ 1g/day	Paracetamol tablets	3 g/day
		Placebo tablets	Inactive placebo
2	Non-opioid analgesics/paracetamol > 1 g/day, and/or NSAID^∗^/or no analgesics – but with difficulty swallowing tablets Buprenorphine 5 mcg/h	Buprenorphine transdermal system	5 μg/h (maximum 10 μg/h)
		Placebo transdermal system	Inactive placebo

*^∗^Except low-dose acetylsalicylic acid.*

Sleep-related outcomes were measured with *Actiwatch Spectrum (Philips Respironics)*. Activity was assessed continuously for 7 days at baseline and for 7 days in week 13 of the treatment period. The intervention started on day 8. Actigraphs were placed on the dominant/mobile wrist. As of today, there is no standard regarding the placement of the actigraph (Camargos et al., 2013). However, in prior studies in which sleep is evaluated with actigraphy, the dominant arm is most commonly used (Camargos et al., 2013). This is based on the understanding that many NH patients may have limited mobility and therefore any potential activity is more likely to occur in the dominant/mobile wrist. NH staff was instructed to push the event button on the actigraph when the patient went to bed in the evening (lights off) and got up in the morning (lights on). These instructions were given both verbally and in writing, and NH staff was provided with contact information if there were any questions regarding this procedure. The *Actiware 6 (Respironics)* was used for sleep scoring. The actigraph’s sensitivity to detect motion was set to medium. Furthermore, sleep/waking status was determined for each one-minute epoch. A qualified technician scored all the activity protocols. A standardized ranked approach was applied to set rest intervals for the actigraphy data, using: event markers when possible, light and activity data, or light or activity data.

The scoring protocol generated data on the following outcome variables: total sleep time (TST), sleep efficiency (SE), sleep onset latency (SOL), wake after sleep onset (WASO), early morning awakening (EMA), and number of wake bouts (NoW). These parameters were estimated in the time window between lights off in the evening and lights on in the morning. In addition, daytime total sleep time (DTS) was estimated in the time window from lights on to lights off using the *Actiware 6* software.

Pain was measured by MOBID-2 (Husebo et al., 2007), a validated, reliable staff-administered instrument with good responsiveness for measuring pain in people with advanced dementia (Husebo et al., 2007, 2014). A total score ranging from 0 to 10 was set, where 10 represented the worst possible pain. The average score was based on all of the observations during the last week. Clinically relevant pain is defined as a score of ≥3 (Husebo et al., 2014).

Symptoms of depression during the last week were assessed using the validated CSDD, an instrument that consists of 19 items measuring five domains related to depression (mood, behavioral disturbances, physical signs, cyclic functions and ideational disturbances). In line with previous research, which has demonstrated that a score of 8/9 complies with the diagnosis of depression according to ICD-10 criteria, the patient had to get a CSDD score ≥ 8 to be included in the study (Barca et al., 2010). The CSDD score was provided using only information from NH staff who knew the patients well.

MMSE was used to evaluate cognitive function. MMSE is a brief, cognitive screening test with a 30-point scale that consists of 20 tasks. It was developed to distinguish potential dementia from normal functioning (Perneczky et al., 2006). Scores from 0 to 10 indicate severe dementia; from 11 to 20 indicate moderate dementia; from 21 to 25 indicate mild impairment; and from 26 to 30 indicate no dementia (Perneczky et al., 2006). A score of ≤20 was necessary to be included in the study.

Initially, 162 patients were included. By means of computer-generated random numbers, these patients were randomly allocated to each arm in a 1:1 ratio. A statistician produced the randomization list without any involvement from the research team. Stratification factors were not used. However, not all of the patients from the main study were included in the actigraphy subproject (see the flow chart in **Figure 1** for the reasons for inclusion/exclusion). The randomization ratio in the actigraphy subproject was therefore not 1:1. The statistician provided the research team with a blinded, sequential list of pack identification numbers, in which patients were consecutively assigned to the next pack number in the list upon inclusion. The study was double-blinded, which implied that all researchers, patients and NH staff were masked with regard to group allocation.

The patient’s medical decision-making capacity was deliberated with the patient’s primary nurse. For patients who had reduced capacity to give consent (MMSE scores from 16 to 19), attempts were made to modify the information. Also, the researchers contacted all of the legal guardians of eligible patients. Legal guardians who gave presumed consent on behalf of the patient received written and oral information together with a consent form to sign and mail back. The *Regional Ethics Committee* (REC-West 2013/1474) approved the study, and the study’s Clinical Trial number is NCT02267057.

### Statistical Analyses

Descriptive statistics (means, standard deviations and percentages) were calculated and compared across the experimental groups both at baseline (week 0) and post-treatment (week 13). In order to investigate the effect of pain treatment after 13 weeks, linear mixed models were conducted. Mixed models allow for regression-based analyses of treatment effects even in the case of considerable attrition, as long as data are missing at random (Bennett, 2001). Thus, individuals with missing data at one time point can be retained in the analyses. The mixed model for the main effect (*n* = 106) was conducted with no covariates, with baseline as the time reference point, and with random intercept. In addition, we conducted a 2 × 2 ANOVA analysis in which we included only data from the 58 patients who completed week 13. This was done to investigate if the different analyses provided similar results.

In addition to the mixed model for the main effect, several *post hoc* sub-group analyses were carried out. Linear mixed model analyses were conducted for the sub-group of patients with MOBID-2 score ≥ 3 (*n* = 46) at baseline and the sub-group of patients with poor sleep at baseline, defined as sleep efficiency < 85% (Lacks and Morin, 1992) (*n* = 90). Finally, linear mixed model analyses were conducted to compare patients receiving active paracetamol and active buprenorphine treatment, respectively (*n* = 57). The statistical analyses were conducted using *IBM SPSS Statistics 24.*

## Results

Two thousand three hundred and twenty three patients were screened for potential inclusion and 106 patients were included in the actigraphy subproject (see flow chart in **Figure 1**). Mean age was 85.5 years and 76% of the patients were female. Mean scores for MMSE, MOBID-2 and CSDD were 7.6, 2.8, and 11.2, respectively (see **Table 2**). From baseline to week 13, 48 patients dropped out of the study (reasons for dropout are listed in **Table 3**). There were no statistically significant differences in relevant baseline characteristics (age, sex, CSDD, MOBID-2, NPI-NH, MMSE) between the patients who dropped out (*n* = 48) and the patients who completed treatment through week 13 (*n* = 58) (see **Table 4**). This supports the assumption that the data were missing at random. Nine patients were using buprenorphine 5 μg/h prior to inclusion and stayed on this treatment and were then randomized to receive either an additional 5 μg/h (5 patients) or placebo patch (4 patients).

**Table 2:** Baseline characteristics for the different treatment groups.

	Placebo group (*n* = 49)	Active group (*n* = 57)	Total (*n* = 106)
Age (mean, *SD*)	86.0 (6.6)	85.2 (7.8)	85.5 (7.3)
Female (%)	80	74	76
MMSE (mean, *SD*)	6.9 (5.8)	8.2 (6.1)	7.6 (6.0)
MOBID-2 (mean, *SD*)	3.2 (2.3)	2.6 (1.9)	2.8 (2.1)
CSDD (mean, *SD*)	11.4 (4.1)	11.0 (3.4)	11.2 (3.7)

*The table reports baseline characteristics for several central variables in the placebo group and the active group: age, sex, mini mental status examination score (MMSE), Mobilization-Observation-Behavior-Intensity-Dementia-2 Pain Scale score (MOBID-2), Cornell Scale for Depression in Dementia score (CSDD). No statistical significant differences were found between the groups*.

**Table 3:** Overview of dropout (*n* = 48) in week 13.

	Placebo tablets (*n* = 2)	Active paracetamol (*n* = 13)	Placebo patch (*n* = 11)	Active buprenorphine (*n* = 22)	All patients (*n* = 48)
Gastrointestinal	0	0	0	4	4
Neurological	0	0	0	2	2
Psychological	0	2	0	6	8
Infection	0	0	1	0	1
Falls/fractures	1	1	0	2	4
No actigraphic measure due to patient refused/malfunction	1	7	8	6	22
Patient refused to take the medication	0	3	0	0	3
Change in treatment	0	0	1	0	1
Death	0	0	1	2	3

*The table reports reasons for dropout in each of the four experimental groups: active/placebo paracetamol/buprenorphine.*

**Table 4:** Comparison of baseline characteristics for patients who completed the treatment and patients who dropped out.

	Completed week 13 (*n* = 58)	Dropout (*n* = 48)	Total (*n* = 106)
Age (mean, *SD*)	84.5 (7.1)	86.8 (7.3)	85.5 (7.3)
Female (%)	78	75	76
MMSE (mean, *SD*)	7.1 (5.7)	8.3 (6.3)	7.6 (6.0)
MOBID-2 (mean, *SD*)	2.8 (2.0)	2.8 (2.3)	2.8 (2.1)
CSDD (mean, *SD*)	11.4 (3.8)	10.9 (3.7)	11.2 (3.7)

*The table reports baseline characteristics for several central variables in the group of patients who completed the study period and those who dropped out; age, sex, MMSE, Mobilization-Observation-Behavior-Intensity-Dementia-2 Pain Scale score (MOBID-2), Cornell Scale for Depression in Dementia score (CSDD). No statistical significant differences were found between the groups*.

The main linear mixed model analyses for sleep outcomes showed no statistically significant differences between patients who received active pain treatment compared to those who received placebo (see **Table 5**). Similarly, in the 2 × 2 ANOVA analyses with only the 58 patients who had complete data in week 13, there were no significant differences between active pain treatment and placebo. **Table 5** also shows descriptive statistics of sleep characteristics for both the placebo group and the active group, as measured at baseline and in week 13 of the treatment period.

**Table 5:** Linear mixed model analyses investigating the long-term effect of pain treatment on sleep outcomes (*n* = 106).

Sleep outcomes		Treatment effect	Pre-post sleep; mean (*SD*)	
			Active	Placebo
TST (min)	*C*	-2.52	515.2 (139.3) – 538.5 (142.4)	509.9 (113.6) – 498.3 (146.8)
	*p*	0.90		
SE (%)	*C*	-0.78	70.0 (15.0) – 73.0 (15.3)	70.0 (13.1) – 68.5 (18.2)
	*p*	0.76		
SOL (min)	*C*	5.68	33.0 (37.3) – 31.3 (43.0)	47.0 (44.5) – 52.2 (63.5)
	*p*	0.61		
WASO (min)	*C*	-7.27	134.5 (66.4) – 116.9 (51.7)	140.6 (68.3) – 133.7 (69.8)
	*p*	0.58		
EMA (min)	*C*	11.73	48.9 (60.4) – 44.4 (54.9)	30.7 (38.9) – 42.8 (45.5)
	*p*	0.22		
NoW (no)	*C*	-0.38	30.3 (12.5) – 28.1 (11.5)	31.2 (11.6) – 30.7 (11.0)
	*p*	0.90		
DTS (min)	*C*	26.27	191.7 (124.0) – 206.2 (130.3)	215.8 (104.2) – 254.5 (106.5)
	*p*	0.15		

*The table reports linear mixed model analyses for the following outcome variables: TST, total sleep time; SE, sleep efficiency; SOL, sleep onset latency; WASO, wake after sleep onset; EMA, early morning awakening; NoW, number of wake bouts; DTS, daytime total sleep time. The column “Treatment effect” reports the interaction effect between treatment and time, i.e., the main result of the clinical trial, with baseline as the reference time point. C refers to coefficients and p refers to p-values. The column “Pre-post sleep; mean (SD)” reports descriptive sleep characteristics for the active and placebo groups from baseline to week 13, with standard deviations in parentheses.*

**Table 6** reports analyses for the subgroup of patients with pain (MOBID-2 score ≥ 3) at baseline. There were no statistically significant differences between the patients who received active treatment and those who received placebo. **Table 7** shows analyses for the subgroup of patients with sleep efficiency < 85% at baseline. Again, there were no statistically significant differences between the patients who received active treatment and those who received placebo.

**Table 6:** Linear mixed model analyses for the subgroup of patients with pain (MOBID-2 score ≥ 3) at baseline (*n* = 46).

Sleep outcomes		Treatment effect	Pre-post sleep; mean (*SD*)	
			Active	Placebo
TST (min)	*C*	-9.71	563.5 (139.1) – 635.1 (152.2)	517.8 (122.2) – 500.8 (151.8)
	*p*	0.80		
SE (%)	*C*	-1.17	75.2 (14.4) – 80.4 (16.0)	70.5 (14.4) – 67.9 (18.4)
	*p*	0.77		
SOL (min)	*C*	7.85	23.5 (25.8) – 17.7 (32.7)	40.5 (43.6) – 49.0 (55.2)
	*p*	0.50		
WASO (min)	*C*	1.12	124.3 (71.2) – 91.7 (61.4)	138.7 (65.5) – 142.1 (73.0)
	*p*	0.97		
EMA (min)	*C*	-1.02	32.2 (35.2) – 43.5 (60.0)	36.6 (49.6) – 43.4 (51.6)
	*p*	0.95		
NoW (no)	*C*	2.03	32.2 (15.8) – 25.3 (16.0)	32.8 (15.2) – 32.5 (13.8)
	*p*	0.75		
DTS (min)	*C*	28.33	251.3 (140.3) – 253.4 (126.5)	226.8 (114.6) – 254.5 (101.6)
	*p*	0.39		

*The table reports linear mixed model analyses for the subgroup of patients with pain (MOBID-2 score ≥ 3) for the following outcome variables: TST, total sleep time; SE, sleep efficiency; SOL, sleep onset latency; WASO, wake after sleep onset; EMA, early morning awakening; NoW, number of wake bouts; DTS, daytime total sleep time. The column “Treatment effect” reports the interaction effect between treatment and time, i.e., the main result of the clinical trial, with baseline as the reference time point. C refers to coefficients and p refers to p-values. The column “Pre-post sleep; mean (SD)” reports descriptive sleep characteristics for the active and placebo groups from baseline to week 13, with standard deviations in parentheses.*

**Table 7:** Linear mixed model analysis for the subgroup of patients with poor sleep (sleep efficiency < 85%) at baseline (*n* = 90).

Sleep outcomes		Treatment effect	Pre-post sleep; mean (*SD*)	
			Active	Placebo
TST (min)	*C*	-4.81	477.1 (116.7) – 463.2 (104.9)	490.2 (95.3) – 470.4 (130.3)
	*p*	0.85		
SE (%)	*C*	-1.54	65.5 (12.5) – 65.7 (13.6)	67.7 (11.0) – 65.2 (16.9)
	*p*	0.64		
SOL (min)	*C*	-0.24	38.7 (38.7) – 45.7 (47.5)	50.1 (43.6) – 58.5 (65.0)
	*p*	0.99		
WASO (min)	*C*	3.93	153.0 (57.5) – 136.4 (40.2)	151.3 (62.5) – 147.1 (64.3)
	*p*	0.81		
EMA (min)	*C*	14.11	56.8 (63.6) – 57.7 (64.1)	33.3 (36.8) – 46.8 (46.8)
	*p*	0.27		
NoW (no)	*C*	-1.58	32.7 (11.1) – 32.0 (10.0)	32.7 (11.1) – 32.0 (12.1)
	*p*	0.61		
DTS (min)	*C*	7.74	161.6 – 167.5 (105.7 – 110.4)	205.2 (98.9) – 240.5 (110.7)
	*p*	0.72		

*The table reports linear mixed model analyses for the subgroup of patients with sleep efficiency < 85% for the following outcome variables: TST, total sleep time; SE, sleep efficiency; SOL, sleep onset latency; WASO, wake after sleep onset; EMA, early morning awakening; NoW, number of wake bouts; DTS, daytime total sleep time. The column “Treatment effect” reports the interaction effect between treatment and time, i.e., the main result of the clinical trial, with baseline as the reference time point. C refers to coefficients and p refers to p-values. The column “Pre-post sleep; mean (SD)” reports descriptive sleep characteristics for the active and placebo groups from baseline to week 13, with standard deviations in parentheses.*

**Table 8** reports analyses for the subgroup of patients receiving the two different types of active pain treatment – paracetamol and buprenorphine, respectively. In this linear mixed model, there were significant effects on TST (*p* < 0.01) and SE (*p* < 0.05), which revealed that TST and SE were both improved after 13 weeks for patients who received active buprenorphine compared with patients who received active paracetamol.

**Table 8:** Linear mixed model analysis for the subgroup of patients receiving the two different types of active pain treatment – paracetamol and buprenorphine (*n* = 57).

Sleep outcomes		Treatment effect	Pre-post sleep; mean (*SD*)	
			Paracetamol	Buprenorphine
TST (min)	*C*	68.56	522.9 (149.1) – 511.4 (141.9)	508.7 (132.6) – 580.6 (140.7)
	*p*	**0.01**		
*SE* (%)	*C*	7.32	71.4 (14.4) – 70.2 (15.0)	68.7 (15.7) – 77.4 (15.8)
	*p*	**0.03**		
SOL (min)	*C*	-20.66	37.9 (40.0) – 41.4 (48.4)	29.0 (35.0) – 15.6 (28.8)
	*p*	0.14		
WASO (min)	*C*	-14.91	121.6 (62.5) – 118.8 (38.4)	145.3 (68.7) – 113.9 (70.3)
	*p*	0.54		
EMA (min)	*C*	-19.93	42.1 (46.7) – 48.2 (57.2)	54.7 (70.1) – 38.5 (54.0)
	*p*	0.26		
NoW (no)	*C*	-10.17	28.0 (11.7) – 30.8 (8.24)	32.2 (13.0) – 23.7 (14.7)
	*p*	0.07		
DTS (min)	*C*	44.04	173.2 (127.0) – 167.1 (124.6)	207.2 (121.3) – 267.0 (120.9)
	*p*	0.10		

*The table reports linear mixed model analyses for the subgroup of patients receiving active paracetamol and active buprenorphine, respectively, for the following outcome variables: TST, total sleep time; SE, sleep efficiency; SOL, sleep onset latency; WASO, wake after sleep onset; EMA, early morning awakening; Now, number of wake bouts; DTS, daytime total sleep time. The column “Treatment effect” reports the interaction effect between type of treatment and time, i.e., the main result of the clinical trial, with baseline as the reference time point. C refers to coefficients and p refers to p-values. P-values printed in bold denote statistical significance. The column “Pre-post sleep; mean (SD)” reports descriptive sleep characteristics for the active and placebo groups from baseline to week 13, with standard deviations in parentheses.*

## Discussion

This is the first placebo-controlled trial to investigate the long-term efficacy of paracetamol and buprenorphine on sleep in patients with comorbid dementia and depression. Previous studies have found that depression among NH patients with dementia may be related to untreated pain (Leong and Nuo, 2007). Moreover, it is well established that pain is associated with sleep disturbances (Chen et al., 2011; Flo et al., 2017). Based on our findings in Blytt et al. (2017a) that pain treatment improved sleep in NH patients with comorbid dementia and depression after one week of pain treatment, we hypothesized that pain treatment would continue to improve sleep after 13 weeks in this patient group. Contrary to our hypothesis, the main mixed model analyses for the full sample showed no statistically significant differences between active and placebo treatment.

There were, however, interesting significant effects in one of the *post hoc* sub-group analyses: TST improved for patients who received active buprenorphine, compared to those who received active paracetamol. In the active paracetamol group, TST was reduced by about 10 min, while it increased by more than one hour in the active buprenorphine group. Furthermore, we found that SE was reduced in the group who received active paracetamol, while it increased by about 9% in the group who received active buprenorphine. Thus, patients who received active buprenorphine seemed to benefit from the treatment. These results are in line with Blytt et al. (2017a), wherein we also found that the group of patients who received active buprenorphine had significantly improved TST compared to the active paracetamol group after one week of treatment. However, the underlying mechanisms are unclear.

Sedation is a frequently reported opioid-associated side effect (McNicol et al., 2003). Usually, symptoms of sedation decline after a few days in more healthy adults. However, among people with comorbidity, sedation may persist (McNicol et al., 2003). In Blytt et al. (2017a), we highlight that the positive effect on TST after one week of treatment could be attributed to such a side-effect. There is a lack of studies that investigate how symptoms of sedation may persist among older people with comorbidity, and we cannot exclude sedation as a potential explanation of the results from the present study.

Importantly, we found no clear causal effect on the active group with clinically significant pain (MOBID-2 score ≥ 3), compared to placebo. This is contrary to previous studies on older people, in which sleep disturbances have been linked to untreated pain (Chen et al., 2011). In addition, pain has previously been shown to reduce SE and to increase WASO and stage 1 sleep at the expense of slow wave sleep and REM sleep (Onen et al., 2005). It is, however, noteworthy that in the subgroup analysis including patients with clinically significant pain, all of the sleep parameters (except EMA) showed indication of improvement, compared to placebo. However, no statistically significant differences were found. This could, however, be attributed to the low number of patients with pain at baseline (*n* = 46), and we cannot exclude type 2 errors.

### Limitations and Strengths

Due to the considerable attrition of patients at week 13, we conducted linear mixed model analyses. These analyses are appropriate to handle missing data and can take into account the dependency of the observations (Bennett, 2001).

There was a drop-out of 22 patients in the group who received active buprenorphine, suggesting that many patients did not tolerate such treatment (see **Table 3**). This large drop-out may have hindered our ability to detect a positive effect from the active treatment compared to placebo. Furthermore, it should be noted that the assignment to paracetamol or buprenorphine was not a result of randomization, but of whether the patients qualified for allocation to either paracetamol or buprenorphine at baseline (and then were randomized to either active or placebo treatment). This may produce bias by indication, since the choice of drug might be related to the outcome.

In addition, during the last decade, there has been a change in the prescription of pain medication for NH patients. Sandvik et al. (2016) found that the use of paracetamol and strong opioids increased significantly from 2000 to 2011. This affected the inclusion of patients, since patients already taking opioids could not be included in the study. Prior to inclusion, nine patients were using buprenorphine, of which five patients received active treatment and four patients received placebo treatment. This is not a source of bias since the comparison was between baseline data (pre-treatment) and week 13 (post-treatment). Therefore, any potential effects measured in week 13 will be additional effects of the treatment. However, the large drop-out in combination with the difficulty to recruit patients to the study is a threat to the generalizability of the study and we cannot exclude selection bias.

Measuring sleep with actigraphy has its limitations. Actigraphy only records movement, and a lack of movement would therefore be assessed as sleep. The study population had low SE, and previous studies show that actigraphy is less accurate in distinguishing sleep from wakefulness when SE is reduced (Sivertsen et al., 2006). Actigraphy recordings may therefore overestimate sleep relative to sleep diaries (Kushida et al., 2001; Sivertsen et al., 2006). It is therefore recommended that clinicians use sleep diaries/logs in addition to actigraphy, when evaluating sleep in NH patients. This would have strengthened the study design.

An additional limitation of the study was that we did not conduct *a priori* power analyses. The lack of this renders us unable to assess whether the statistical analyses had sufficient power. It is, however, noteworthy that our sample of patients with actigraphy was similar or larger than samples in comparable studies (Fetveit and Bjorvatn, 2005; Dowling et al., 2008; Camargos et al., 2014).

Compared to placebo, pain treatment did not improve sleep in the full sample of patients, as measured with actigraphy. However, we found a significant effect on TST and SE, when we compared the different types of active pain treatment. These results indicate that some patients may experience beneficial effects of pain treatment. However, the underlying mechanisms are unclear. The results could be an indication that some of the patients in fact experience pain, and hence had a positive effect of more potent pain treatment. Future research should investigate this further, with a larger sample size and including patients with clinically significant pain.

## Author Contributions

KB designed the study, analyzed the data, and wrote the paper. BB, EF, and BH designed the study, helped with the analysis of the data, and wrote the paper.

## Conflict of Interest Statement

Mundipharma International supplied the study medication, but the company did not influence the study design, data collection, analyses and interpretation of data, or final publication. The authors declare that the research was conducted in the absence of any commercial or financial relationships that could be construed as a potential conflict of interest. The reviewer JV declared a past co-authorship with one of the authors BH.

## References

[B1] American Psychiatric Association (ed.). (2013). *Diagnostic and Statistical Manual of Mental Disorders* 5th Edn Arlington, VA: American Psychiatric Association 10.1176/appi.books.9780890425596

[B2] Barca M. L. Engedal K. Selbæk G. (2010). A reliability and validity study of the Cornell scale among elderly inpatients, using various clinical criteria. *Dement. Geriatr. Cogn. Disord.* 29 438–447. 10.1159/000313533 20502018

[B3] Bennett D. A. (2001). How can I deal with missing data in my study? *Aust. N. Z. J. Public Health* 25 464–469. 10.1111/j.1467-842X.2001.tb00294.x 11688629

[B4] Blytt K. M. Bjorvatn B. Husebo B. Flo E. (2017a). Effects of pain treatment on sleep in nursing home patients with dementia and depression – a multicentre placebo- controlled randomised clinical trial. *Sleep Med.* 40:e39. 10.1002/gps.4839 29282768 PMC5873424

[B5] Blytt K. M. Selbæk G. Drageset J. Natvig G. K. Husebo B. S. (2017b). Comorbid dementia and cancer in residents of nursing homes: secondary analyses of a cross-sectional study. *Cancer Nurs.* 10.1097/NCC.0000000000000478 [Epub ahead of print]. 28146014 PMC5839697

[B6] Camargos E. F. Louzada F. M. Nóbrega O. T. (2013). Wrist actigraphy for measuring sleep in intervention studies with Alzheimer’s disease patients: application, usefulness, and challenges. *Sleep Med. Rev.* 17 475–488. 10.1016/j.smrv.2013.01.006 23669093

[B7] Camargos E. F. Louzada L. L. Quintas J. L. Naves J. O. Louzada F. M. Nóbrega O. T. (2014). Trazodone improves sleep parameters in Alzheimer disease patients: a randomized, double-blind, and placebo-controlled study. *Am. J. Geriatr. Psychiatry* 22 1565–1574. 10.1016/j.jagp.2013.12.174 24495406

[B8] Chen Q. Hayman L. L. Shmerling R. H. Bean J. F. Leveille S. G. (2011). Characteristics of chronic pain associated with sleep difficulty in older adults: the maintenance of balance, independent living, intellect, and zest in the elderly (MOBILIZE) Boston study. *J. Am. Geriatr. Soc.* 59 1385–1392. 10.1111/j.1532-5415.2011.03544.x 21806564 PMC3307096

[B9] Chopra K. Arora V. (2014). An intricate relationship between pain and depression: clinical correlates, coactivation factors and therapeutic targets. *Expert Opin. Ther. Targets* 18 159–176. 10.1517/14728222.2014.855720 24295272

[B10] Cricco M. Simonsick E. M. Foley D. J. (2001). The impact of insomnia on cognitive functioning in older adults. *J. Am. Geriatr. Soc.* 49 1185–1189. 10.1046/j.1532-5415.2001.49235.x 11559377

[B11] Dew M. A. Hoch C. C. Buysse D. J. Monk T. H. Begley A. E. Houck P. R. (2003). Healthy older adults’ sleep predicts all-cause mortality at 4 to 19 years of follow-up. *Psychosom. Med.* 65 63–73. 10.1097/01.PSY.0000039756.23250.7C 12554816

[B12] Dowling G. A. Burr R. L. Van Someren E. J. Hubbard E. M. Luxenberg J. S. Mastick J. (2008). Melatonin and bright-light treatment for rest–activity disruption in institutionalized patients with Alzheimer’s disease. *J. Am. Geriatr. Soc.* 56 239–246. 10.1111/j.1532-5415.2007.01543.x 18070004 PMC2642966

[B13] Fetveit A. Bjorvatn B. (2005). Bright-light treatment reduces actigraphic-measured daytime sleep in nursing home patients with dementia: a pilot study. *Am. J. Geriatr. Psychiatry* 13 420–423. 10.1176/appi.ajgp.13.5.420 15879592

[B14] Flo E. Bjorvatn B. Corbett A. Pallesen S. Husebo B. S. (2017). Joint occurrence of pain and sleep disturbances in people with dementia. A systematic review. *Curr. Alzheimer Res.* 14 538–545. 10.2174/1567205013666160602234932 27335036

[B15] Giron M. S. Forsell Y. Bernsten C. Thorslund M. Winblad B. Fastbom J. (2002). Sleep problems in a very old population: drug use and clinical correlates. *J. Gerontol. A Biol. Sci. Med. Sci.* 57 M236–M240. 10.1093/gerona/57.4.M236 11909889

[B16] Helvik A. S. Engedal K. Benth J. Š. Selbæk G. (2015). Prevalence and severity of dementia in nursing home residents. *Dement. Geriatr. Cogn. Disord.* 40 166–177. 10.1159/000433525 26138271

[B17] Husebo B. S. Ballard C. Fritze F. Sandvik R. K. Aarsland D. (2013). Efficacy of pain treatment on mood syndrome in patients with dementia: a randomized clinical trial. *Int. J. Geriatr. Psychiatry.* 29 828–836. 10.1002/gps.4063 24806873

[B18] Husebo B. S. Ostelo R. Strand L. I. (2014). The MOBID-2 pain scale: reliability and responsiveness to pain in patients with dementia. *Eur. J. Pain* 18 1419–1430. 10.1002/ejp.507 24799157 PMC4230478

[B19] Husebo B. S. Strand L. I. Moe-Nilssen R. Husebo S. B. Snow A. L. Ljunggren A. E. (2007). Mobilization-observation-behavior-intensity-dementia pain scale (MOBID): development and validation of a nurse-administered pain assessment tool for use in dementia. *J. Pain Symptom Manage.* 34 67–80. 10.1016/j.jpainsymman.2006.10.016 17509814

[B20] Husebo B. S. Strand L. I. Moe-Nilssen R. Husebo S. B. Ljunggren A. E. (2010). Pain in older persons with severe dementia. Psychometric properties of the mobilization–observation–behaviour–intensity–dementia (MOBID-2) pain scale in a clinical setting. *Scand. J. Caring Sci.* 24 380–391. 10.1111/j.1471-6712.2009.00710.x 20210897

[B21] Jacobs D. Ancoli-Israel S. Parker L. Kripke D. F. (1989). Twenty-four-hour sleep-wake patterns in a nursing home population. *Psychol. Aging* 4 352–356. 10.1037/0882-7974.4.3.352 2803629

[B22] Kinnunen K. M. Vikhanova A. Livingston G. (2017). The management of sleep disorders in dementia: an update. *Curr. Opin. Psychiatry* 30 491–497. 10.1097/YCO.0000000000000370 28858007

[B23] Kushida C. A. Chang A. Gadkary C. Guilleminault C. Carrillo O. Dement W. C. (2001). Comparison of actigraphic, polysomnographic, and subjective assessment of sleep parameters in sleep-disordered patients. *Sleep Med.* 2 389–396. 10.1016/S1389-9457(00)00098-8 14592388

[B24] Lacks P. Morin C. M. (1992). Recent advances in the assessment and treatment of insomnia. *J. Consult. Clin. Psychol.* 60 586–594. 10.1037/0022-006X.60.4.586 1506506

[B25] Leong I. Y. Nuo T. H. (2007). Prevalence of pain in nursing home residents with different cognitive and communicative abilities. *Clin. J. Pain* 23 119–127. 10.1097/01.ajp.0000210951.01503.3b 17237660

[B26] Lyketsos C. G. Olin J. (2002). Depression in Alzheimer’s disease: overview and treatment. *Biol. Psychiatry* 52 243–252. 10.1016/S0006-3223(02)01348-3 12182930

[B27] McCleery J. Cohen D. A. Sharpley A. L. (2016). Pharmacotherapies for sleep disturbances in dementia. *Cochrane Database Syst. Rev.* 11:CD009178. 10.1002/14651858.CD009178.pub3 27851868 PMC6464889

[B28] McNicol E. Horowicz-Mehler N. Fisk R. A. Bennett K. Gialeli-Goudas M. Chew P. W. (2003). Management of opioid side effects in cancer-related and chronic noncancer pain: a systematic review. *J. Pain* 4 231–256. 10.1016/S1526-5900(03)00556-X 14622694

[B29] Montplaisir J. Petit D. Gauthier S. Gaudreau H. Decary A. (1998). Sleep disturbances and EEG s lowing in Alzheimer’s disease. *Sleep Res. Online* 1 147–151. 11382871

[B30] Montplaisir J. Petit D. Lorrain D. Gauthier S. Nielsen T. (1995). Sleep in Alzheimer’s disease: further considerations on the role of brainstem and forebrain cholinergic populations in sleep-wake mechanisms. *Sleep* 18 145–148. 10.1093/sleep/18.3.145 7610309

[B31] Moran M. Lynch C. A. Walsh C. Coen R. Coakley D. Lawlor B. A. (2005). Sleep disturbance in mild to moderate Alzheimer’s disease. *Sleep Med.* 6 347–352. 10.1016/j.sleep.2004.12.005 15978517

[B32] Morley J. E. (2013). Frailty, falls, and fractures. *J. Am. Med. Dir. Assoc.* 14 149–151. 10.1016/j.jamda.2012.12.009 23375679

[B33] Neikrug A. B. Ancoli-Israel S. (2010). Sleep disturbances in nursing homes. *J. Nutr. Health Aging* 14 207–211. 10.1007/s12603-010-0051-8 20191255 PMC12878877

[B34] Onen S. H. Onen F. Courpron P. Dubray C. (2005). How pain and analgesics disturb sleep. *Clin. J. Pain* 21 422–431. 10.1097/01.ajp.0000129757.31856.f7 16093748

[B35] Ownby R. L. Peruyera G. Acevedo A. Loewenstein D. Sevush S. (2014). Subtypes of sleep problems in patients with Alzheimer disease. *Am. J. Geriatr. Psychiatry* 22 148–156. 10.1016/j.jagp.2012.08.001 23567445

[B36] Pat-Horenczyk R. Klauber M. R. Shochat T. Ancoli-Israel S. (1998). Hourly profiles of sleep and wakefulness in severely versus mild-moderately demented nursing home patients. *Aging Clin. Exp. Res.* 10 308–315. 10.1007/BF03339793 9825022

[B37] Perneczky R. Wagenpfeil S. Komossa K. Grimmer T. Diehl J. Kurz A. (2006). Mapping scores onto stages: mini-mental state examination and clinical dementia rating. *Am. J. Geriatr. Psychiatry* 14 139–144. 10.1097/01.JGP.0000192478.82189.a8 16473978

[B38] Peter-Derex L. Yammine P. Bastuji H. Croisile B. (2015). Sleep and Alzheimer’s disease. *Sleep Med. Rev.* 19 29–38. 10.1016/j.smrv.2014.03.007 24846773

[B39] Potter G. G. Steffens D. C. (2007). Contribution of depression to cognitive impairment and dementia in older adults. *Neurologist* 13 105–117. 10.1097/01.nrl.0000252947.15389.a9 17495754

[B40] Prince M. Comas-Herrera A. Knapp M. Guerchet M. Karagiannidou M. (2016). *World Alzheimer Report 2016. Improving Healthcare for People Living with Dementia. Alzheimer’s Disease International*. Available at: https://www.alz.co.uk/research/worldalzheimerreport2016sheet.pdf [accessed April 20, 2017].

[B41] Sandvik R. Selbaek G. Kirkevold O. Husebo B. S. Aarsland D. (2016). Analgesic prescribing patterns in Norwegian nursing homes from 2000 to 2011: trend analyses of four data samples. *Age Ageing* 45 54–60. 10.1093/ageing/afv184 26764395

[B42] Sivertsen B. Omvik S. Havik O. E. Pallesen S. Bjorvatn B. Nielsen G. H. (2006). A comparison of actigraphy and polysomnography in older adults. *Sleep* 29 1353–1358. 10.1093/sleep/29.10.1353 17068990

[B43] Stone K. L. Blackwell T. Ancoli-Israel S. Redline S. Claman D. (2004). Impaired sleep increases the risk of falls in older woman: a prospective actigraphy study. *Sleep* 27:A125.

[B44] Widera E. (2013). What’s to blame for falls and fractures? Poor sleep or the sleeping medication? Comment on “Nonbenzodiazepine sleep medication use and hip fractures in nursing home patients”. *JAMA Intern. Med.* 173 761–762. 10.1001/jamainternmed.2013.3801 23459766

